# Poisoning by Strychnia

**Published:** 1869-02

**Authors:** A. M. Carpenter

**Affiliations:** Professor of the Institutes and Practice of Medicine, Medical Department Iowa State University


### POISONING BY STRYCHNIA.

BY A. M. CARPENTER, M. D.,

Professor of the Institutes and Practice of Medicine, Medical Department Iowa State University.

About 3 o'clock A. M., on the 15th of October, 1857, I received a hurried summond to visit Mr. Jno. L. W. Alt, aged 22 years, who was supposed by the messenger to be suffering from a "violent fit." On repairing to the apartment indicated, the first feature of attraction was the peculiar expression of terror in the young man's countenance. Approaching the bed side and having touched the pulse, I was startled to observe that the mere contact of my index was a signal, as it were, to electrify his whole frame with one of the severest clonic spasms, with complete opisthotonic contraction of the cervical muscles, that I ever witnessed. Instituting close inquiry, I gathered the fact that he had quarrelled with his brother, and smarting under the remorse which it engendered, was induced to terminate his own life by a resort to the sulphate of strychnia, which he had purchased ostensibly "to kill a dog." The amount, I learned subsequently, was about three grains. In a few moments after swallowing the powder, he experienced a cramping or twisting sensation in the stomach, which rapidly propagated itself to the muscles of the superior extremities, including those of the neck and spine. The convulsive phenomena were akin to those movements exhibited during an epileptoid seizure—no frothing at the mouth nor evidences of asphyxia. The hands were thrown upwards to grasp the slats in the head of the bed, while the tonic contraction was strenuous enough to shiver them to pieces. So terrific were the tetanic efforts and so extreme the suffering, that no matter how steeled a physician might be, he could but share in the unutterable distress of his patient. The duration of the tetanic spasm in the muscles of the arms and back was about thirty seconds, and the *interim* betwixt the efforts from two to three minutes; the pulse feeble and frequent; skin cool, except the face, which was slightly flushed; throat dry; voice husky, and responses to questions disjointed; pupils moderately contracted. Having no knowledge (of an agent capable of producing a chemical change in poison) at that day, the first indication was to evacuate the stomach, which was promptly effected by pulvis ipecac, grs. 30; tartrate antimony, grs. 2, dissolved in a pint of water and administered in large spoonful doses every few minutes. The spasmodic recurrences seemed delayed a little after free emesis had taken place—a ray of encouragement to be sure. Meantime, a messenger was dispatched for a bottle of chloroform, the vapor of which he greedily inhaled for the space of half an hour—the spasms recurring at longer intervals and marked with less severity than before the vapor was applied, while fewer of the muscles participated in the convulsive effort, until, in the course of an hour, he became almost tranquil, save slight tetanic twitching when contact was practiced upon any part of the body. It may be remarked that the vapor was not pushed to the production of complete Anesthesia. The general muscular rigidity having lessened, the patient was enjoined to seek repose under the calmative influence of half grain doses of sulph. morph., to be repeated every thirty minutes. Returning in three hours, found the paient tranquil; had slept at short intervals, interrupted only by occasional starting. The morphia was continued at intervals of an hour, with instructions to withhold it in case he slept soundly.

In twenty-four hours the patient took some panada, and signified his desire to get up and dress himself, feeling no greater inconvenience than some lurking stiffness of the cervical and spinal muscles and the flexures of the joints. Grateful for his happy deliverance, and sorely penitent at his own folly, he was admonished "to go and sin no more."

The persistent use of emetics in the foregoing case contributed largely to the fortunate issue, though had the patient not been accessible to prompt medical aid, he must surely have perished. While the emetic served to eliminate some portion of the toxic salt, yet to the anti-spasmodic influence of chloroform and morphia the happy termination was mainly due.

September 24th, 1863, I was requested to see Miss A. J. S., aged 27 years, a stranger sojourning in this city, who voluntarily swallowed a portion of strychnia, supposed to be about *two grains*, dissolved in a teaspoonful of plain water. I ascertained that a physician had been sent for as soon as the discovery was made, and that chloroform had been ordered for the purpose of subduing the spasmodic action which was becoming, as I learned, "more violent within the last hour." The evacuation of the contents of the stomach—so indispensible as a prelude to the successful management of strychnia poisoning—having been overlooked, and the space of *four* hours having elapsed from the time of taking the salt until my arrival, I could hope for no benefit from an emetic after almost complete absorption of the poison had taken place, as evidenced by the violent and oft-recurring spasmodic action. Remembering the experiments of Prof. Kurzak of Germany upon dogs and rabbits—after taking the drug, as detailed in the American Journal of Medical Sciences, number for January, 1863, with *tannin* in doses twenty times greater than the amount of the poison swallowed—I at once resolved upon the experiment, and hence prescribed one dram of tannin to be divided into six powders, of which she took one every fifteen minutes, sprinkled upon the tongue and washed down with cold water. At the end of an hour the patient could speak intelligibly, the severity of the contractions having sensiby abated. As in the foregoing case, the touch of the wrist or the slightest movement of the limbs would evoke the most fearful tetanic effort, which was confined to the upper and lower extremities, in which latter the cramps remained after the convulsive effort ceased. During or towards the close of a spasm, the pulse would intermit, the respiratory action be almost suspended, and the eye glare as if in "articulo mortis;" feet and surface became cool; the capillaries seemed to be emptied, and it was not without effort, externally applied, that re-action was awakened. The tannin was applied vigorously, until she had taken *seventy* grains. (No chloroform was exhibited by my direction, nor had she inhaled a full dram by direction of the previous attendant.) The spasms growing less and less, she was ordered forty drops of liquor morphia comp. every twenty minutes; after the lapse of an hour she became tranqnil, except when the wrist was felt. The sedative influence of the morphia was kept up at longer intervals during the evening, but failed to subdue that morbid hyperesthesia of the nerve centres to a degree sufficient to produce sleep, though the night was passed in comparative comfort.

September 25th, visiting my patient, I was informed by the nurse that she would frequently exclaim through the night that she would "soon be dead." In reply to a question the patient stated that she had taken the poison and would "certainly die." No confession as to the motive which impelled her to the rash act could be extorted from her. It was however evident that she was laboring under a fit of jealousy, or some domestic infelicity.

The symptoms present were great thirst, furred tongue, dry in the centre, taste disordered, moderate febrile movement, pulse 120, general soreness of muscles, tenderness over stomach with nausea, vomiting and constipation. She was ordered eight grains Sub. Mur. Hyd., with a quarter of a grain $\overline{aa}$. of ipecac and morphia, to be followed by a saline cathartic, which served to depurate the secretory system generally; the urgent symptoms subsiding, though the stomach remained irritable for several days, and yielded ultimately to vesication and the endermic use of morphia, with small bits of ice internally to allay thirst and precordial heat. She took only wine jelly as nourishment, and was discharged on the 2d of October, and left the city.

I may venture the opinion that the favorable termination in this case may be ascribed to the antidotal properties of the tannic acid; the resultant effect would seem at least analogous in the human subject to that attained by the experiments upon the canine species, as detailed by the German professor. No explanation has been offered as to the chemical change wrought by the introduction of tannin. As an agent capable of neutralizing the toxic effects of one of the most dangerous articles known to Materia Medica, we must accord to it certain antidotal powers. Morphia and its salts rank high as an important auxiliary in these cases, in subduing the convulsions and cramps and conducing to the relief of the suffering consequent upon these manifestations, as well as an anti-phlogistic of no feeble power in combatting the gastric excitement that may ensue, the dose of which should be measured by the exigencies of individual cases. Dr. John Bartlett, of this city, informs me of the good effect of common salt in arresting spasm in dogs poisoned by strichnia, used in doses of a large teaspoonful every few minutes. In every instance, if early called, the stomach should be emptied; tannin in large doses, chloroform by inhalation at intervals, and lastly morphia to subdue the spasmodic twitching, and the remote effects of the drug, should be boldly practiced.

